# An *in silico* evaluation of treatment regimens for recurrent *Clostridium difficile* infection

**DOI:** 10.1371/journal.pone.0182815

**Published:** 2017-08-11

**Authors:** Natalia Blanco, Betsy Foxman, Anurag N. Malani, Min Zhang, Seth Walk, Alexander H. Rickard, Marisa C. Eisenberg

**Affiliations:** 1 Department of Epidemiology, School of Public Health, University of Michigan. Ann Arbor, Michigan, United States of America; 2 Department of Infection Prevention & Control, Department of Internal Medicine, Division of Infectious Diseases, St. Joseph Mercy Health System, Ann Arbor, Michigan, United States of America; 3 Department of Biostatistics, School of Public Health, University of Michigan, Ann Arbor, Michigan, United States of America; 4 Department of Microbiology and Immunology, College of Letters & Science, Montana State, Bozeman, Montana, United States of America; Cleveland Clinic, UNITED STATES

## Abstract

**Background:**

*Clostridium difficile* infection (CDI) is a significant nosocomial infection worldwide, that recurs in as many as 35% of infections. Risk of CDI recurrence varies by ribotype, which also vary in sporulation and germination rates. Whether sporulation/germination mediate risk of recurrence and effectiveness of treatment of recurring CDI remains unclear. We aim to assess the role of sporulation/germination patterns on risk of recurrence, and the relative effectiveness of the recommended tapered/pulsing regimens using an *in silico* model.

**Methods:**

We created a compartmental in-host mathematical model of CDI, composed of vegetative cells, toxins, and spores, to explore whether sporulation and germination have an impact on recurrence rates. We also simulated the effectiveness of three tapered/pulsed vancomycin regimens by ribotype.

**Results:**

Simulations underscored the importance of sporulation/germination patterns in determining pathogenicity and transmission. All recommended regimens for recurring CDI tested were effective in reducing risk of an additional recurrence. Most modified regimens were still effective even after reducing the duration or dosage of vancomycin. However, the effectiveness of treatment varied by ribotype.

**Conclusion:**

Current CDI vancomycin regimen for treating recurrent cases should be studied further to better balance associated risks and benefits.

## Introduction

*Clostridium difficile* is an anaerobic, spore-forming, Gram-positive bacillus associated with the toxin-mediated intestinal disease known as *C*. *difficile* infection (CDI)[1, 2]. Over the last two decades, CDI morbidity and mortality has increased in all five continents [3]. CDI treatment mostly involves a course of oral metronidazole or vancomycin [4, 5]. Recurrence, defined as a subsequent CDI within 8 weeks following resolution of the initial episode [6], occurs in 5–35% of patients following appropriate treatment [7–9].

*C*. *difficile* spores are resistant to therapy, so those remaining after treatment can germinate and lead to recurrence [10]. Ribotypes with higher sporulation rates, for example ribotype 027, are associated with higher rates of recurrence [11, 12]. Similarly, strains with high germination efficiency are associated with severe and recurrent CDI [13, 14].

In order to encourage spores to germinate and become vulnerable to therapy, tapering or pulsing of oral vancomycin is recommended for treating recurrent—particularly repeated recurrent—CDI [4]. The regimen also allows the microbiota to recover [15, 16]. Although clinical trials show tapered/pulsed vancomycin treatments are more effective at reducing CDI recurrence than the standard longer and higher doses [16], no controlled data exist evaluating the relative effectiveness of specific tapering and pulsing regimens [17].

Mathematical models provide a method for comparing the relative effectiveness of different regimens in the absence of a controlled trial. We present a mathematical model to simulate the levels of spores and vegetative cells within the CDI host by the four most common ribotypes in the U.S. [18]. Using this *in silico* model, we compared the importance of *C*. *difficile* sporulation/germination patterns in selected ribotypes, and estimated the contribution of sporulation/germination patterns to observed differences in CDI recurrence rates. In addition, we evaluated the effectiveness of current tapered/pulsed vancomycin regimens for recurring CDI by ribotype.

## Methods

### Deterministic ordinary differential equation (ODE) model

We developed a compartmental in-host mathematical model for CDI patients, composed of the major parts of the bacteria’s life cycle within the human host. As our purpose was to evaluate CDI recurrence, our model simulated and measured: number of vegetative cells (C), germinating spores (Spl), non-germinating spores (Spd), and toxin (T) per mL of gut contents per day. We note that under optimal circumstances almost all spores may germinate and hence are not technically ‘non-germinating,’ but for ease of presentation we use ‘non-germinating’ to designate spores that do not germinate under the gut conditions simulated here.

Our model is described with the following equations:

$$\begin{array}{l} \frac{dC}{dt}=kC\left(1-\frac{C^{3}}{Cap^{3}}\right)+k_{ger}Spl-k_{txt}CV-(k_{ExC}+k_{sp})C\\ \frac{dSpd}{dt}=(1-SpV)(k_{sp}C)-(k_{ExSpd}Spd)\\ \frac{dSpl}{dt}=SpV(k_{sp}C)-(k_{ExSpl}+k_{ger})Spl\\ \frac{dT_{1}}{dt}=k_{tox}C-k_{ExT}T\\ \frac{dT_{2}}{dt}=k_{ExT}(T_{1}-T_{2})\\ \frac{dV}{dt}=u(t)-k_{V}V,\;where\;u(t)=vancomycin\;input \end{array}$$

#### Vegetative cell and vancomycin equations

Our first equation represents *C*.*difficile* vegetative cells (C). Vegetative cells are able to proliferate in the colon if conditions permit; however, a protective microbiota and other processes may inhibit colonization [7]. When modeling the growth of *C*. *difficile* vegetative cells (C), we first considered the bacteria’s growth rate (k) limited by their carrying capacity within the human gut (Cap). For the logistic growth term, we tested several exponents and chose the lowest integer value that yielded a visually good fit (cubic power). In addition, we considered the formation of new cells due to the germination of available spores (k_ger_). We also subtracted the loss of cells (k_LC_), either because they sporulated (k_sp_) or they were shed into the environment through defecation (k_ExC_).

Finally, we considered the loss or inactivation of cells due to vancomycin treatment (k_txt_). To better represent the vancomycin pharmacokinetics, we added an extra equation to our model. When using the standard regimen of 125mg/L four times a day, oral vancomycin is poorly absorbed, so stool concentrations significantly exceed the MIC_90_ of most *C*. *difficile* isolates [4, 19]. The vancomycin concentration (V) was first fitted to vancomycin data [20] using sum of least of squares, then its parameter (k_v_) was fixed for the remainder of the parameter fitting.

#### Spores/toxin equations

During its life cycle, *C*. *difficile* vegetative cells produce endospores [4]. Spores are highly resistant to the immune system, antibiotics, and harsh environmental conditions [1, 21]. If ingested, spores survive the stomach’s acid environment and germinate into vegetative cells in the small intestine when stimulated by bile salts [1, 21]. Vegetative cells of toxigenic strains produce several toxins. Toxin A and toxin B are most commonly associated with *C*. *difficile* associated disease [1].

In order to account for different degrees of sporulation varibility across ribotypes, we separated spores into two compartments: germinating (Spl) and non-germinating (Spd). SpV represents the fraction of germinating spores produced by ribotype. We further accounted for the *C*. *difficile* sporulation rate (k_*sp*_) and the loss of spores (k_LS_) either because they are shed into the environment through feces (k_ExSpl_/k_ExSpd_) or they germinate into vegetative cells (k_ger_).

Similarly, in the toxin compartment, we accounted for the toxin production rate by vegetative cells (k_tox_) and exit of toxin (k_ExT_), either because it was used up, lost through feces, or decayed. We also incorporated a toxin delay, by a series of stages (n) through which the toxin had to pass before exiting the toxin compartment. The latter allowed us to account for the slow decay of the toxin in the gut/feces [22]. We chose the smallest number of compartments that yielded the best fit, which resulted in n = 2.

#### Parameter estimation and sensitivity analysis

To streamline model development and parameter estimation, we used a forcing function approach (similar to that developed in [23] and commonly used in glucose-insulin models and other physiological models[24, 25]) in which the overall model was broken into two submodels (S1 and S2 Figs) to generate initial ‘ballpark’ parameter estimates, and then recombined for final parameter estimation using the complete model. This approach allowed us to separately evaluate the level of simplification needed for the model structure in our initial model explorations. Parameters were estimated using *C*. *difficile* ribotype 027 data from a gut laboratory model reported by Baines et al. 2009 [20], using least squares. We then evaluated the parameter uncertainty using Fisher information-based confidence intervals (using the Cramér-Rao bound), which we report as percent coefficient of variation (%CV), defined as 100 times the standard deviation (SD) of the parameter, divided by the parameter estimate [23, 26, 27]. Further details of the forcing function approach used and the sensitivity analysis performed can be found in the online supplement (S1 File). All model simulations and parameter estimation was performed in MATLAB (2015b), with parameter estimation via least squares using *fminsearch*.

**Fig 1:**
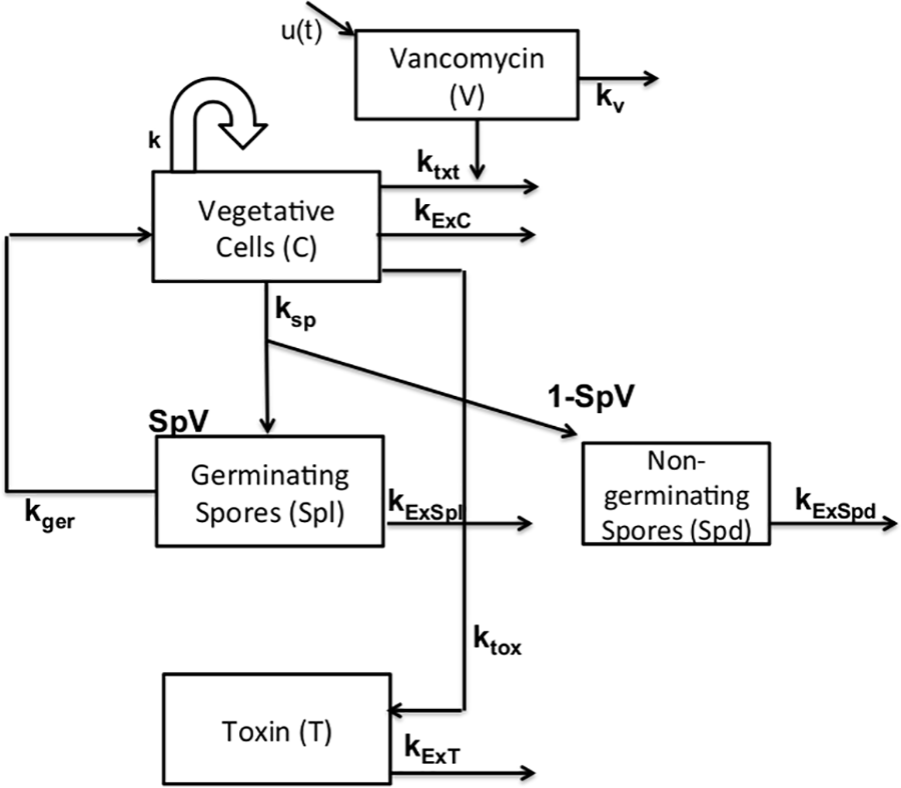
Graphical representation of the overall in-host compartmental CDI model within its human host.

In addition, although the human host is not an explicit set of compartments or variables in our model, we have defined it as an in-host mathematical model, as the host is implicit in the data used for fitting, the parameter values chosen, and the simulated treatment regimes (as described below). However, we are unable to distinguish in our model between different hosts, as we do not account for intra-host variability. Our complete in-host mathematical model is graphically represented in Fig 1 and S3 Fig. Parameter details for ribotype 027 can be found in Table 1.

**Table 1:** Final overall model parameters for ribotype 027, as fitted to data from Baines et al. 2009 [20]*.

Model Parameter	Description	Value	%CV**
N	Number of toxin delay compartments	2	–
K	*C*. *difficile* growth rate (cells/day)	1.1953	2.9
Cap	Carriage capacity (cells/day)	1.2241x10^6^	0.94
k_sp_	Sporulation rate (1/day)	0.0072	2.4
SpV	Fraction of germinating spores (1/day)	0.534050	92.8
k_ger_	Germination rate (1/day)	0.0006	105.7
k_tox_	Toxin production rate (1/day)	0.0043	9.6
k_ExC_	Exit rate of vegetative cells (1/day)	0.0352	97.0
k_ExSpd_	Exit rate of non-germinating spores (1/day)	0.3577	36.8
k_ExSpl_	Exit rate of germinating spores (1/day)	0.3197	30.2
K_ExT_	Exit rate of toxin (1/day)	0.9491	7.5
K_txt_	Treatment killing rate (vc killed/day)	1.5811	78.8
k_v_	Exit rate of vancomycin (1/day)	1.3116	120.7
u(t)	Vancomycin input	Simulation dependent	–

*These parameters were used to feed our simulations for all ribotypes, the sporulation rates (k_sp_) and fraction of germinating spores (SpV) were the only parameters that were modified based on the ribotype across our model simulations.

**For the fitted parameters, the percent coefficient of variation is given (%CV = 100 x SD/parameter value).

### Stochastic model

An ODE model can only provide an average of recurrence by ribotype, and stochasticity may play an important role during extinction or recurrence of an infection. Thus, we created a stochastic model based on our ODE model to estimate the probability of recurrence by ribotype. We focused on three compartments of the ODE model for stochastic simulation: 1) vegetative cells, 2) non-germinating spores, and 3) germinating spores. As vancomycin concentrations are continuous values and unlikely to be stochastic at the scale we are considering, we used the same ODE representation for vancomycin treatment as in the deterministic model. We simulated the vancomycin concentration by treatment regimen ahead of time using a simple model, which only included the vancomycin equation. The resulting datasets were used to feed our stochastic model.

Due to the large number of vegetative cells and spores that made up our system, we simulated our model using the Tau-leaping method [28]. This method determines the probability of each event to happen over each pre-specified interval of time, in our case every 2.4 hours (10 times a day). The model simulated the probability of occurrence of 5 specific events: 1) vegetative cell growth, 2) germination of spores, 3) exit or death of vegetative cells, 4) sporulation of vegetative cells, and 5) exit of germinating and non-germinating spores. After each evaluation, the model added or subtracted vegetative cells or spores from their specific compartment as needed. At the end of the simulation, we compiled the total number of vegetative cells and spores.

### Simulations

#### Effect of sporulation rates and viability of spores on CDI recurrence

We based our simulations on the most prevalent ribotypes currently circulating in the US: ribotypes 027, 002, 014–020, and 106 [18]. Using data from the literature (Table 2) and the fitted parameters of our overall model of ribotype 027 (Table 1), we estimated an average rate of sporulation and a fraction of germinating spores for each ribotype. For example, using *in vitro* data from the literature, we estimated an average number of spores produced per day per mL of 40,295 for ribotype 002, while for 027 we estimated 109,054 spores per day per mL. As we knew the sporulation rate of 027 from our model fit, we were able to solve for an approximate sporulation rate of ribotype 002 (k_sp002_ = k_sp027_/(109,054/40,295). Similarly, we solved for the k_sp_ of the other ribotypes. The fractions of germinating spores per ribotype were averaged from the literature [13, 29–34]. The parameters are described in Table 2.

**Table 2:** Sporulation rates, spore germinability, and data source by selected ribotypes used to inform the in-host model of *Clostridium difficile* infection.

Simulation Parameters			
**Sporulation rates by ribotype**	**Spores/day per mL**	**Sporulation rate ((spores/day)/mL)**	**Sources used**
027-based parameters	109,054		[29, 31, 32, 34, 35]
002- based parameters	40,295	0.002665654	[31, 32]
106- based parameters	184,555	0.012208954	[31, 32]
014–020 based parameters	2,773	0.000183444	[29]
**Spore germinability by ribotype**	**Fraction of spore viability**		**Sources used**
027- based parameters	0.5341		[13, 29, 33]
002- based parameters	0.4700		[33]
106- based parameters	0.4151		[13]
014–020 based parameters	0.6276		[29, 33]

We ran both of our models for a total of 200 days. All model parameters remained the same as in Table 1, except for sporulation rates and spore viability, which we modified by ribotype (Table 2). On day 13, we added the regular vancomycin treatment (125mg/L four times a day for 10 or 14 days) to our simulation. The literature suggests a 13 day-period provides the necessary time for the pathogen’s vegetative cells, spores, and toxin to reach the required levels to initiate symptoms within the host [20]. The stochastic model was run 500 times for each ribotype.

#### Vancomycin tapered/pulsed-treatment effectiveness on CDI recurrence

**Fig 2:**
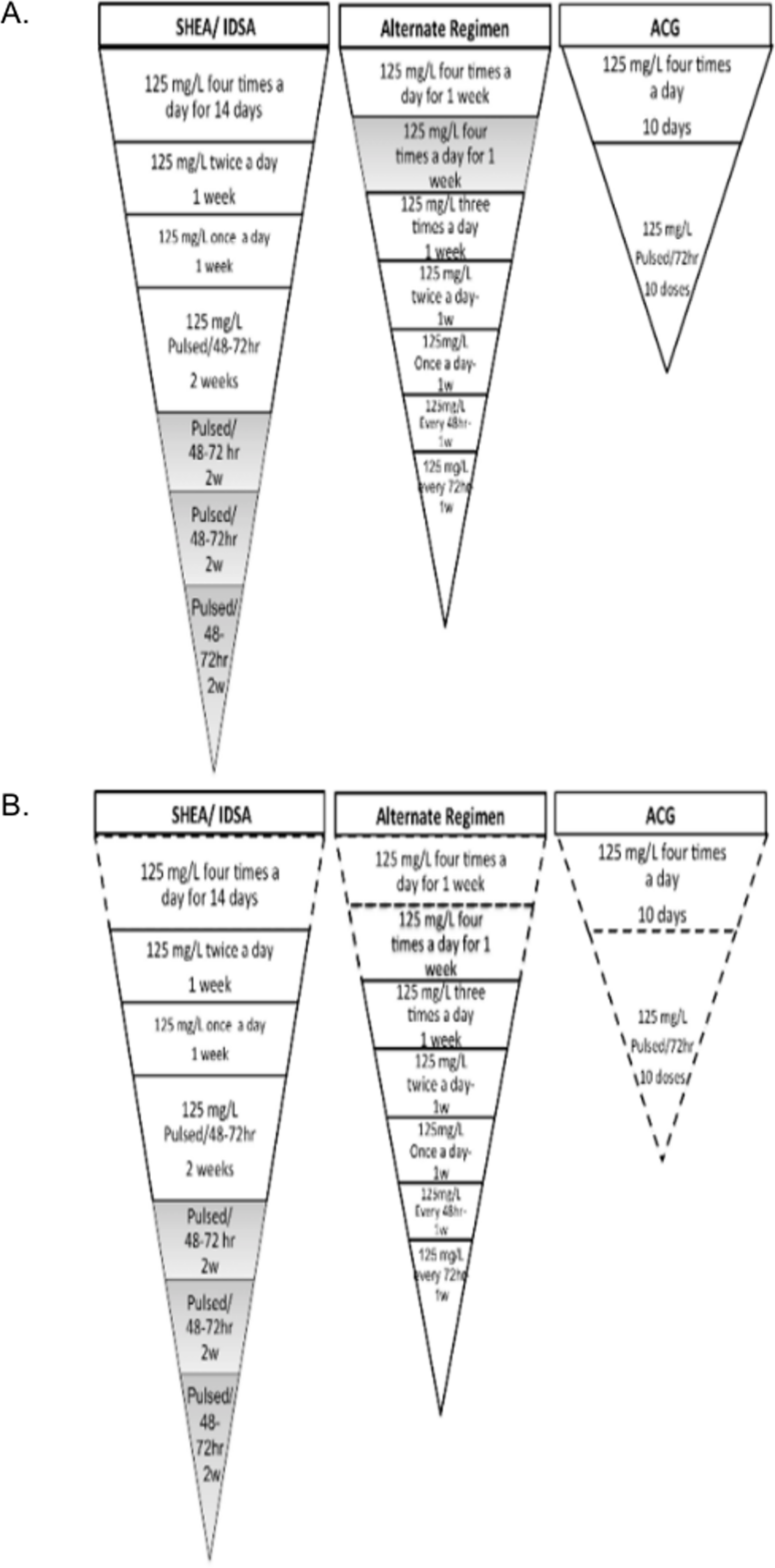
Graphical representation of the simulated tapered/pulsed oral vancomycin regimens. (A)Recommended tapered/pulsed oral vancomycin regimens. (B) Modified recommended regimens. We tested three specific regimens: one recommended by The Society for Healthcare Epidemiology of America (IDSA/SHEA), another by the American College of Gastroenterology (ACG), and the third one is an alternate regimen based on expert opinion [5]. The gray-colored blocks represent optional steps, and the dotted-lined blocks in panel (B) represent the steps that were eliminated or modified for the purposes of this study. Abbreviations: w = week, hr = h.

On day 13, we simulated three commonly used tapered/pulsed vancomycin treatments (Fig 2). The Infectious Diseases Society of America/ Society for Healthcare Epidemiology of America (IDSA/SHEA) 2010 recommendations for recurrent CDI include oral vancomycin 125mg/L four times daily for 10–14 days (we simulated 14 days instead of 10, in order to be more conservative), followed by vancomycin twice a day for a week, then vancomycin once daily for one week, concluding with vancomycin every 48–72 hours for 2–8 weeks [4]. Similarly, the College of Gastroenterology (ACG) recommends 125 mg/L 4 times a day for 10 days followed by every 72 hours for ten doses [17]. Finally, a recent published review recommends (based on expert opinion) an alternate regimen described as follows: 125mg/L four times daily for 1–2 weeks, then 125mg/L three times a day for 1 week, followed by 125mg/L twice a day for 1 week, then 125mg/L once daily for 1 week, next 125mg/L every 48 hours for 1 week, and concluding with 125mg/L every 72 hours for 1 week [5].

In addition, we further tested modifications on these commonly used regimens. We tested the SHEA/IDSA recommended regimens omitting the initial regular treatment (125 mg/L four times a day for 14 days). Similarly, we experimented with the alternate regimen removing the initial regular treatment of oral vancomycin four times a day for 1–2 weeks. We further tested a modified ACG regimen by 1) reducing the number of pulsed doses and 2) reducing the vancomycin dose from 125mg/L 4 times a day to twice a day (Fig 2).

For the ODE model, due to the lack of stochasticity, any amount of cell or spore material (even if much less than a single cell) will eventually “recur”, even though this would be unrealistic in real-world settings. Thus, for these simulations we also modified our ODE models to indicate a minimal threshold of 0.01% of one cell/spore for the vegetative cell or spore compartments for recurrence. The stochastic model was run 500 times for each ribotype per regimen.

## Results

Our in-host mathematical model simulates the pathogenicity and transmission patterns of *C*. *difficile* within its human host (Fig 1 and S4 Fig). The final model parameters are described in Tables 1 and 2. The germination and vancomycin exit rates had %CVs > 100% (105.7% and 120.7% respectively) indicating relatively large uncertainty, but all parameters were identifiable [36].

### Simulations

#### Effect of sporulation rates and viability of spores on CDI recurrence

**Fig 3:**
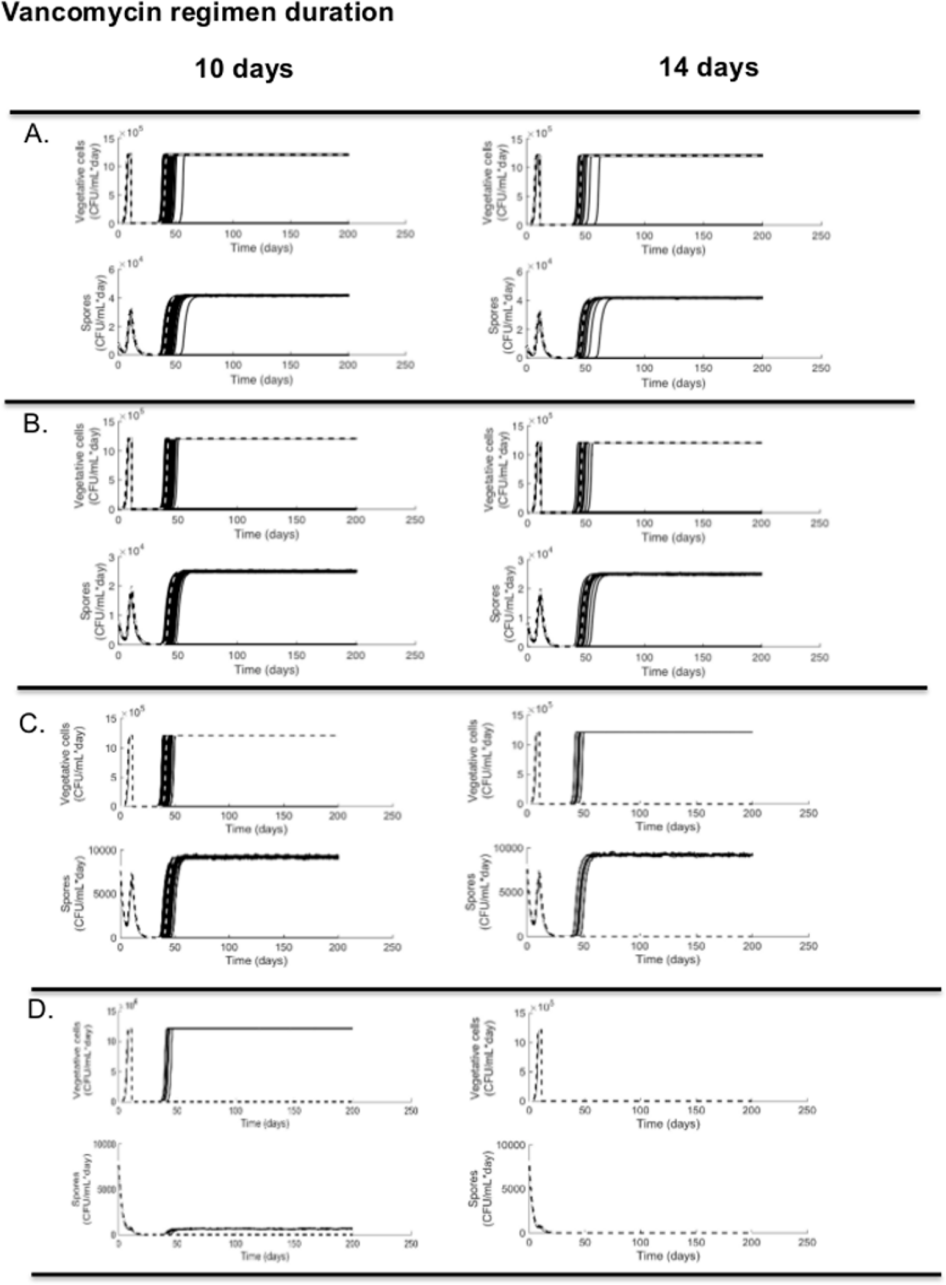
Simulated recurrence rate by ribotype: Differences on sporulation rates and fraction of germinating spores per ribotype are reflected on differences on recurrence rates. (A)Simulates ribotype 106 based parameters (4.0–18.8% recurrence). (B) Simulates ribotype 027 based parameters (4.0–12.2% recurrence).(C) Simulates ribotype 002 based parameters (1.2–6.8% recurrence). (D) Simulates ribotype 014–020 based parameters (0–1.0% recurrence). On day 13 post exposure, we added to the model the regular vancomycin CDI treatment: 125mg/L four times a day for 10 days (left) or 125mg/L four times a day for 14 days (right). The black solid lines represent each of the 500 runs of the stochastic model, and the white slashed-line represents the results of our deterministic model.

As expected, sporulation rates and viability of spores affected the pathogenicity and transmission patterns in our simulation. Using the deterministic model, all ribotypes but 014–020 recurred after 10 days of 125 mg/L four times a day. Ribotype 014–020 was able to cause a first CDI episode, but initial treatment was sufficient to avoid recurrence. By contrast, ribotypes (027, 106, 002) recurred approximately 30–55 days after initial CDI treatment. However, when we extended the regular treatment to 14 days, ribotype 002 also did not recur (Fig 3).

In our stochastic model, all of the ribotypes recurred in at least one simulation after 10 days of treatment. However, the probability of recurrence varied by ribotype: ribotype 106 (18.8%), ribotype 027 (12.2%), ribotype 002 (6.8%), and 014–020 (1.0%). This matches the more binary results seen in the deterministic model, with a more nuanced outcome. Similarly, when we extended treatment 4 extra days (14 days total), the probability of recurrence was reduced even further: ribotype 106 (4.0%), ribotype 027 (4.0%), ribotype 002 (1.2%), and 014–020 (0%) (Fig 3).

#### Vancomycin tapered/pulsed-treatment effectiveness on CDI recurrence

For all ribotypes, all recommended vancomycin tapered/pulsed regimens were effective in avoiding recurrence following repeated infection. (Table 3). When we completely eliminated the initial regular treatment (125mg/L four times a day for 14 days) from the SHEA/IDSA regimen, this regimen remained effective for all ribotypes after only four weeks of pulsed doses. When further modified to only two weeks of pulsing every 48 hours, the SHEA/IDSA regimen remained effective for all ribotypes but 027 (027 recurred 0.2% of the time). Interestingly, if we pulsed every 72 hours for 2 weeks, both ribotype 027 and ribotype 106 recurred but at low levels (0.2–0.6%). Similarly, when we eliminated the 2 initial weeks of 125mg/L of oral vancomycin four times daily from the alternate regimen, we did not observe any recurrences for any of the ribotypes. When we reduced the ACG pulsed doses to 7 (every 72 hours), the regimen remained effective at avoiding recurrence from all ribotypes. Furthermore, when we reduced the dose to 125mg/L twice a day for 10 days followed by pulses every 72 hours for 10 doses; the treatment remained effective, with only ribotype 27 recurring, but at a very low rate (0.2% recurrence) (Table 3).

**Table 3:** Recurrence rate by ribotype after receiving recommended or modified oral vancomycin tapered/pulsed regimens.

Regimen	Ribotypes			
	106	027	002	014–020
*Commonly used regimens*				
SHEA/IDSA: pulses every 48h for 2 weeks	0%	0%	0%	0%
SHEA/IDSA: pulses every 72hr for 2 weeks	0%	0%	0%	0%
Alternate regimen: only 1 week of 125 mg/L four times a day	0%	0%	0%	0%
ACG: Full regimen	0%	0%	0%	0%
*Modifications*				
SHEA/IDSA: no initial regular txt (125 mg/L four times a day for 14 days) + 72 hr pulses for 2 weeks	0.6%	0.2%	0%	0%
SHEA/IDSA: no initial regular txt (125 mg/L four times a day for 14 days) + 48 hr pulses for 2 weeks	0%	0.2%	0%	0%
SHEA/IDSA: no initial regular txt (125 mg/L four times a day for 14 days) + 72 hr pulses for 4 weeks	0%	0%	0%	0%
SHEA/IDSA: no initial regular txt (125 mg/L four times a day for 14 days) + 48 hr pulses for 4 weeks	0%	0%	0%	0%
Alternate regimen: no initial regular treatment (125 mg/L four times a day for 2 weeks)	0%	0%	0%	0%
ACG: 125mg/L twice a day for 10 days + 10 pulsed doses	0%	0.2%	0%	0%
ACG: 125 mg/L 4 times a day for 10 days + only 7 pulsed doses	0%	0%	0%	0%

#### Sensitivity analysis

As discussed in the supplement, the vancomycin killing rate parameter is somewhat uncertain due to the doses used in the experimental data (which are significantly greater than the MIC_90_ for *C*. *difficile*). To assess the sensitivity of the results to the vancomycin treatment parameter, we re-ran the same simulations modifying the vancomycin killing rate across a range of values that yielded approximately the same fit to the data (described in the supplement). This yielded nearly identical results (recurrence rates with the alternate values were all within 2.5% of the recurrence rates using the fitted value of k_txt_).

## Discussion

We used a compartmental in-host mathematical model for CDI patients to simulate *in vivo* toxin and spore rates for the most prevalent *C*. *difficile* ribotypes and evaluate whether ribotype-specific sporulation/germination patterns affect CDI recurrence and effectiveness of treatment regimens for reducing risk of repeated CDI. All recommended treatment regimens were effective in reducing risk of an additional recurrence. Furthermore, reducing the duration or dosage of most of the assessed regimens for tapered/pulsed vancomycin treatment did not change effectiveness.

Our simulations also support previous evidence that differences in sporulation/germination patterns across *C*. *difficile* ribotypes are risk factors for recurrence [12–14]. Ribotype 014–020 has higher spore viability but lower sporulation rates than the other ribotypes evaluated, and recurred up to 1.0% of the time following initial CDI treatment with vancomycin, much less frequently than other ribotypes. Indeed, although this ribotype does recur, it accounts for only 5% of CDI recurrences in North America [37]. Moreover, these results suggest that the CDI recurrence rate can be at least partially explained by differences in sporulation and germination patterns by ribotype, which also validates the model’s predictive ability. In addition, our model highlights the benefit of a longer regular CDI treatment, 14 days instead of 10 days, in order to reduce the likelihood of an initial CDI recurrence.

Using the reported distribution of ribotypes found in hospital and community-associated CDI cases [18] and our recurrence rates, ribotypes 027, 002, 014–020, and 106 would lead to a combined recurrence rate of 3–11% (depending on the duration of regular or initial treatment), which overlaps with the recurrence range of 5–35% reported in the literature [7–9]. Note that the 5–35% range includes both relapses and reinfections by the same or different strains, and our estimate includes only relapses from the same strain. Relapses reportedly account for 25 to 87.5% of all recurrences [14] (i.e. there is a ~1–31% rate of relapse among initial CDI cases), which is also consistent with our results.

In our models, all recommended treatment regimens for repeated CDI recurrence were highly effective in avoiding an additional recurrence, which is validated by the fact that only up to 6% of CDI cases recurred 2 or more times [38]. Our models also suggest that there is no significant difference in treatment efficacy between pulsing periods of 48 or 72 hours when applied to the regimens as they are currently recommended. However, the regimens could be reduced in duration or in dosage and still be highly effective.

This finding has potentially important clinical implications, as vancomycin therapy is not without risk. Vancomycin treatment suppresses *Bacteriodes* spp., a marker of normal gut microbiota [39], and a healthy gut microbiota prevents the introduction or colonization of pathogens, including the reemergence of *C*. *difficile* [40]. Furthermore, vancomycin and similar anti-anaerobic therapy can promote the selection and spread of vancomycin-resistant enterococcus colonization [40, 41]. Our results suggest that vancomycin regimens might be further modified to a level that better protects gut microbiota while preventing CDI recurrence, although this can only be definitely answered using appropriate controlled clinical trials. In addition, studies examining the role of probiotics in conjunction with tapered/pulsed vancomycin may uncover potential regimens to further reduce vancomycin dosage and duration.

Although it is not current clinical practice to report *C*.*difficile* ribotype when CDI is diagnosed in the laboratory, this information could provide valuable additional information. Our model results support the potential of tailoring the initial regular CDI treatment based on the causative *C*. *difficile* ribotype. Patients diagnosed as having CDI due to ribotype 027 are more susceptible to recur; thus, physicians may decide to follow these patients more closely to identify early CDI recurrences. Moreover, these patients may benefit from an initial tapered/pulse oral vancomycin treatment instead of the standard, high dose, initial CDI treatment.

As with any simulation study, our model is limited by the current state of knowledge and the data available to inform the model. *C*. *difficile* vegetative cells, spores and even toxin are found among asymptomatic individuals [42]; thus our model could not distinguish between *C*. *difficile* colonization and symptomatic CDI recurrence. In addition, there is strong evidence supporting the importance of host factors—including gut microbiota and immune system—on CDI development and recovery. However, there is limited *in vivo* human data on the direct effects of these host factors on *C*. *difficile* sporulation and toxin production; because of this, we did not include these factors in the model influencing our simulations towards the pathogen’ side. Due to the negative impact of antibiotic treatment on the gut microbiota diversity and recovery, this limitation may lead to an underestimation of recurrence rates and overestimation of the effectiveness of the treatment [43, 44]. Similarly, our model does not account for biofilm formation within the human gut, which could potentially decrease CDI treatment effectiveness and increase CDI recurrence [45]. Furthermore, the lack of standardization across the methods currently used to measure sporulation/germination rates by ribotype limits our ability to characterize these patterns for each common ribotype. In addition, our model does not account for inter-strain variation of sporulation and germination patterns across ribotypes [20, 31–33, 46, 47]. Inter-host variability is also not taken into account in our model. Finally, our simulations did not include other potential recommended treatments for recurrent CDI, such as fidaxomicin[5].

In conclusion, we developed a compartmental in-host mathematical model of CDI. To our knowledge, this is the first modeling study to examine treatment effects particularly within-host; other published models have examined *C*. *difficile* transmission, pathogenesis, or treatment effectiveness from a different perspective [48–52]. Our results highlight the importance of sporulation/germination patterns across *C*. *difficile* ribotypes on CDI pathogenicity and transmission, which directly affects CDI treatment and infection control. Current CDI vancomycin regimens particularly for treating recurrent cases should be further studied to better balance their associated risks and benefits.

## Supporting information

S1 File Additional details on methodology. Further details on the forcing function approach and sensitivity analysis performed.
(PDF)

S1 Fig Vegetative cell submodel. (TIF)

S2 Fig Spore and toxin submodel. (TIF)

S3 Fig Graphical representation of the overall in-host compartmental CDI model within its human host. Upper rectangle (dotted-lines) represents the vegetative cells submodel. Lower rectangle (dashed-lines) represents the spore/toxin submodel.
(TIF)

S4 Fig Fitting of overall in-host compartmental model of CDI within the symptomatic host (Ribotype 027). Circles indicate the data used for fitting and solid line is the fitted model.
(TIF)
