# Supplementary material for: An in silico evaluation of treatment regimens for recurrent Clostridium difficile infection
Source: PLoS One. 2017 Aug 11;12(8):e0182815. doi: 10.1371/journal.pone.0182815 (PMC5553947; doi:10.1371/journal.pone.0182815)
Supplement: S1 File — Further details on the forcing function approach and sensitivity analysis performed. (PDF) [file pone.0182815.s001.pdf]

S1 File: Additional details on methodology

Forcing function approach

To streamline model development and parameter estimation, we used a forcing function approach (similar to that developed in [1], and commonly used in glucose-insulin models and other physiological models [2, 3]). This general approach initially breaks the overall model into two (or more) submodels, where the links between submodels are measured outputs (i.e. quantities for which we have time course data). Initial parameter estimates are then generated using the submodels, after which the overall model is recombined and overall parameter estimation proceeds as usual using these initial guesses[1-3]. This approach is often useful for facilitating initial model simplification/development, as each submodel can be adjusted to capture the correct input-output behavior, without the effects of the other (potentially also misspecified) submodels affecting the dynamics. Once these initial explorations are complete, the model-building and estimation process typically proceeds as usual[1-3].

The two submodels used in this case were: (1) vegetative cells (Figure 1S) and (2) spores and toxin (Figure 2S). Vegetative cell data (interpolated linearly) was used as an input to the spore and toxin model, while vancomycin concentration and spore data was used as an input to the vegetative cell model. Thus, each submodel could be developed and fit separately. Once this initial development phase was completed, we combined the submodels for final parameter estimation from data. All models were fitted using *C. difficile* ribotype 027 data from a gut laboratory model reported by Baines et al. 2009 [4], using least squares. For the model inputs, we used linear splines to interpolate between data points, generating a continuous input to each submodel.

As mentioned above, a final fit of the overall model was then generated, using the parameter estimates from the forcing function submodels as initial parameter values for the fit (following the approach in [1-3])(Figure 3S and 4S). We then evaluated the parameter uncertainty using Fisher information-based confidence intervals (using the Cramér-Rao bound), which we report as percent coefficient of variation (%CV), defined as 100 times the standard deviation (SD) of the parameter, divided by the parameter estimate [1, 5, 6].

Sensitivity analysis

The recommended vancomycin regimen of 125mg/L four times a day achieves vancomycin fecal levels several hundred times higher than the vancomycin MIC_{90} for *C. difficile* [7]. Thus, our model's ability to estimate the vancomycin-killing rate is limited, as the vancomycin regimen kills all *C. difficile* vegetative cells very quickly. In order to assure a proper fitting of this parameter, we ran our stochastic model using an upper (1.8) and lower threshold (1.3) of k_{txt} values. We chose these thresholds because they were the largest and smallest values that yielded a visually nearly indistinguishable fit to the data. We ran our simulations 1000 times with these values to validate our model and demonstrate its agreement with available literature on recurrence rates.

References:

1. Eisenberg M, Samuels M, DiStefano JJ. Extensions, Validation, and Clinical Applications of a Feedback Control System Simulator of the Hypothalamo-Pituitary-Thyroid Axis. Thyroid. 2008;18(10):1071-85.
2. Dalla Man C, Camilleri M, Cobelli C. A system model of oral glucose absorption: validation on gold standard data. IEEE Trans Biomed Eng. 2006;53(12 Pt 1):2472-8. doi: 10.1109/TBME.2006.883792. PubMed PMID: 17153204.
3. Dalla Man C, Rizza RA, Cobelli C. Meal simulation model of the glucose-insulin system. IEEE Trans Biomed Eng. 2007;54(10):1740-9. doi: 10.1109/TBME.2007.893506. PubMed PMID: 17926672.
4. Baines SD, O'Connor R, Saxton K, Freeman J, Wilcox MH. Activity of vancomycin against epidemic Clostridium difficile strains in a human gut model. The Journal of antimicrobial chemotherapy. 2009;63(3):520-5. doi: 10.1093/jac/dkn502. PubMed PMID: 19112083.
5. Jacquez JA, Perry T. Parameter estimation: local identifiability of parameters. Am J Physiol Endocrinol Metab. 1990;258:E727-E36.
6. Landaw EM, DiStefano JJ. Multiexponential, multicompartmental, and noncompartmental modeling. II. Data analysis and statistical considerations. . Am J Physiol Regul Integr Comp Physiol 1984;246:R665-R77.
7. Gonzales M, Pepin J, Frost EH, Carrier JC, Sirard S, Fortier LC, et al. Faecal pharmacokinetics of orally administered vancomycin in patients with suspected Clostridium difficile infection. BMC infectious diseases. 2010;10:363. doi: 10.1186/1471-2334-10-363. PubMed PMID: 21192802; PubMed Central PMCID: PMCPMC3022836.
