# Supplementary figures and images for: An in silico evaluation of treatment regimens for recurrent Clostridium difficile infection
Source: PLoS One. 2017 Aug 11;12(8):e0182815. doi: 10.1371/journal.pone.0182815 (PMC5553947; doi:10.1371/journal.pone.0182815)

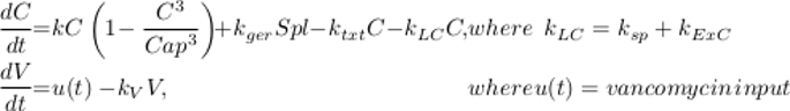

Supplement: S1 Fig — (TIF) [file pone.0182815.s002.tif]

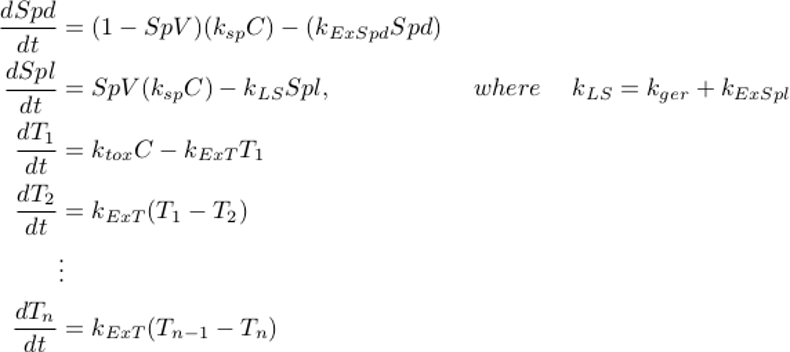

Supplement: S2 Fig — (TIF) [file pone.0182815.s003.tif]

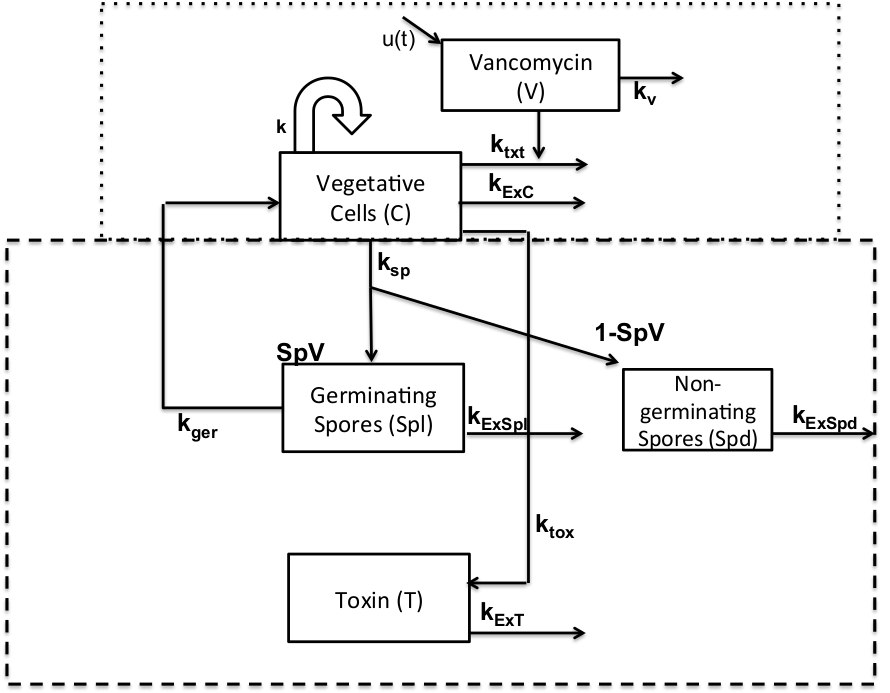

Supplement: S3 Fig — Upper rectangle (dotted-lines) represents the vegetative cells submodel. Lower rectangle (dashed-lines) represents the spore/toxin submodel. (TIF) [file pone.0182815.s004.tif]

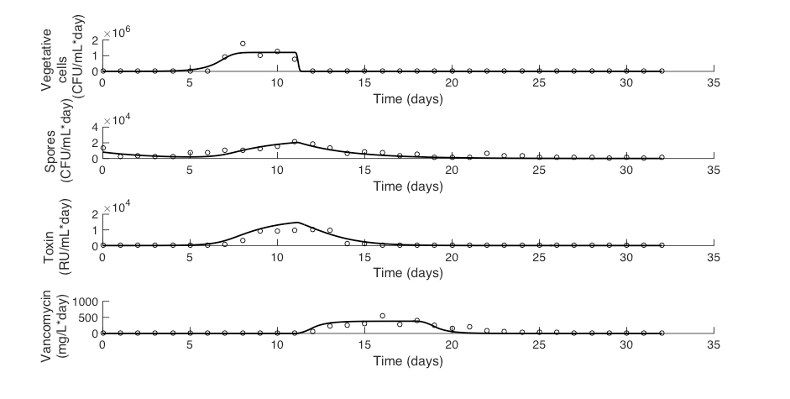

Supplement: S4 Fig — Circles indicate the data used for fitting and solid line is the fitted model. (TIF) [file pone.0182815.s005.tif]
